# Diagnóstico micológico de paracoccidioidomicosis en un hospital de área no endémica: metodología clásica y molecular

**DOI:** 10.7705/biomedica.6888

**Published:** 2023-08-31

**Authors:** Norma B. Fernández, Adriana Toranzo, Luciana Farias, Cristina E. Canteros

**Affiliations:** 1 Laboratorio de Micología, Hospital de Clínicas “José de San Martín”, Universidad de Buenos Aires, Ciudad Autónoma de Buenos Aires, Argentina Universidad de Buenos Aires Universidad de Buenos Aires Ciudad Autónoma de Buenos Aires Argentina; 2 Servicio Micosis Profundas, Departamento de Micología, INEI-ANLIS “Dr. Carlos G. Malbrán”, Ciudad Autónoma de Buenos Aires, Argentina Departamento de Micología, INEI-ANLIS “Dr. Carlos G. Malbrán” Ciudad Autónoma de Buenos Aires Argentina; 3 Departamento de Micología, INEI-ANLIS “Dr. Carlos G. Malbrán”, Ciudad Autónoma de Buenos Aires, Argentina Departamento de Micología, INEI-ANLIS “Dr. Carlos G. Malbrán” Ciudad Autónoma de Buenos Aires Argentina

**Keywords:** Paracoccidioides, paracoccidioidomicosis, técnicas de diagnóstico molecular, ADN, micosis, Paracoccidioides, paracoccidioidomycosis, molecular diagnostic techniques, DNA, mycoses

## Abstract

**Introducción.:**

La paracoccidioidomicosis es una micosis sistémica y endémica en Latinoamérica. El cambio climático y el movimiento migratorio del huésped enfatizan la necesidad de optimizar el diagnóstico de esta infección.

**Objetivo.:**

Evaluar la implementación de la detección de ADN de *Paracoccidioides* spp. al diagnóstico micológico de pacientes con sospecha de paracoccidioidomicosis.

**Materiales y métodos.:**

Estudio retrospectivo con datos de laboratorio de pacientes con sospecha de paracoccidioidomicosis en un hospital de área no endémica.

**Resultados.:**

Se analizaron los resultados de las muestras de 19 pacientes con sospecha clínica de paracoccidioidomicosis. El 90 % de los pacientes había nacido o visitado un área endémica de esta micosis en Latinoamérica. En 14 pacientes varones adultos se confirmó paracoccidioidomicosis por diagnóstico convencional. El examen directo fue positivo en 12 pacientes con enfermedad comprobada y en 4 de ellos se obtuvo crecimiento del hongo. Se detectaron anticuerpos contra *Paracoccidioides* spp. en ocho pacientes con la enfermedad. Se realizó PCR anidada con muestras de 14 pacientes para detectar ADN de *Paracoccidioides* spp. En 9 de los 10 pacientes con diagnóstico convencional de paracoccidioidomicosis se obtuvo una prueba de PCR positiva.

**Conclusiones.:**

La implementación de técnicas moleculares para detectar ADN de *Paracoccidioides* spp. complementa el diagnóstico convencional de paracoccidioidomicosis y permite instaurar el tratamiento antifúngico, sobre todo en los casos clínicos donde no se observa la presencia del hongo en las muestras clínicas. La migración actual de poblaciones humanas dificulta el diagnóstico de paracoccidioidiomicosis y otras infecciones endémicas, por lo que se requiere optimizar el diagnostico micológico en los laboratorios clínicos para tratar pacientes con este tipo micosis desatendida.

## Abstract (en)

**Introduction.:**

Paracoccidioidomycosis is a systemic mycosis endemic in Latin America. Climate change and host migration emphasize the need to optimize this infection diagnosis.

**Objective.:**

To evaluate the implementation of *Paracoccidioides* spp. DNA detection in the mycological diagnosis of patients with suspected paracoccidioidomycosis.

**Materials and methods.:**

It is a retrospective study with laboratory data from patients with clinical suspicion of paracoccidioidomycosis, who consulted a university hospital from a non-endemic area.

**Results.:**

We analyzed the laboratory results of samples from 19 patients with suspected paracoccidioidomycosis. Seventeen out of 19 patients were born in or had visited an endemic area in Latin America. Fourteen adult male patients were confirmed to have paracoccidioidomycosis by conventional diagnosis: the direct examination was positive in 12 samples while fungal growth was found only in 4. Anti-*Paracoccidioides* spp. antibodies were detected in 10 patients, 8 of them with proven paracoccidioidomycosis. Nested PCR for *Paracoccidioides* spp. detection was performed on clinical samples from 14 patients, and positive results were obtained for 9 out of 10 patients with the conventional diagnosis of paracoccidioidomycosis.

**Conclusions.:**

The incorporation of molecular techniques to detect *Paracoccidioides* spp. DNA complements the conventional diagnosis of paracoccidioidomycosis. This tool allows the prescription of antifungal treatment in those cases where the fungus is not observed in the clinical samples. Current human migrations difficult the mycological diagnosis of paracoccidioidomycosis and other fungal infections. For this reason, it is necessary to improve mycological diagnosis in clinical laboratories to adequately treat patients with this neglected mycosis.

La paracoccidioidomicosis es una micosis endémica y sistémica, de distribución geográfica limitada a Latinoamérica, producida por especies de *Paracoccidioides*. Se considera una enfermedad desatendida, cuyo verdadero impacto está poco definido debido a la falta de datos de su incidencia real. Probablemente esto se deba a los pocos estudios epidemiológicos que discuten las formas clínicas, las manifestaciones relacionadas con la diversidad filogenética de las especies, la escasez de diagnóstico oportuno y la falta del reporte obligatorio de casos al sistema de salud regional ^1^^-^^3^.

Durante el 2022, *Paracoccidioides* spp. fue incorporado por la Organización Mundial de la Salud a la lista de agentes patógenos de prioridad moderada para fomentar su investigación y acciones en salud pública ^4^.

El diagnóstico de paracoccidioidiomicosis se basa en los hallazgos clínicos, epidemiológicos y de laboratorio. Es importante hacer el diagnóstico diferencial de esta micosis con otras enfermedades que presentan características clínicas similares y que en algunos casos se presentan como coinfecciones ^5^^-^^7^. La detección del ADN de *Paracoccidioides* spp. brinda resultados rápidos y precisos, y podría incorporarse como herramienta diagnóstica al laboratorio de micología clínica ^8^.

## Antecedentes epidemiológicos, clínicos y de laboratorio

La paracoccidioidomicosis fue reconocida en 1908 como una enfermedad mucocutánea crónica. En 1928, en Brasil, se denominó al agente etiológico de esta enfermedad como *Paracoccidioides* y en 1930 como *Paracoccidioides brasiliensis*. Durante ese año, en Argentina, Pablo Negroni reportó 50 pacientes con paracoccidioidomicosis y demostró *in vitro* el dimorfismo de este hongo. Desde entonces se reportan casos de paracoccidioidomicosis en Latinoamérica, principalmente en Brasil, Colombia, Argentina, México, Paraguay, Bolivia, Ecuador, Venezuela y Perú. ^9^^-^^12^.

En las últimas décadas, se observaron cambios en las características demográficas y la distribución geográfica de la paracoccidioidomicosis. Estos cambios podrían atribuirse al impacto de factores ambientales tales como el cambio climático, la tala de bosques, el aumento de la producción de diversos cultivos en Latinoamérica, la expansión de los asentamientos urbanos y la urbanización de áreas rurales y la presencia de comorbilidades e inmunosupresión de la población, todo esto sumado a la optimización de los métodos de diagnóstico micológico ^13^^,^^14^.

En Latinoamérica, Brasil se destaca por reportar el 80 % de los casos de paracoccidioidomicosis, con una incidencia anual estimada de 3 a 4 casos por millón de habitantes ^9^; y en áreas hiperendémicas, de 90 a 400 casos por millón de habitantes. En el estado de Rondônia, se reportaron 52,7 casos por año, y en la cuenca de Tocantins-Araguaia, 47,1 casos por año. ^15^^-^^20^.

En Argentina, el área endémica de paracoccidioidomicosis está localizada al norte del paralelo 34° S. En el noreste se registra el 85 % de los casos y en el noroeste, los casos restantes. Luego de la construcción de la represa hidroeléctrica Yaciretá, en el 2009, en el noreste del país se alteraron las condiciones ecológicas del valle del río Paraná. Esto se tradujo en cambios climáticos y antropogénicos que impactaron las tasas de paracoccidioidomicosis de los habitantes de la región, en especial, la de la forma infanto-juvenil diagnosticada en el 26 % de los casos de la región norte del país. ^1^^,^^15^^,^^21^.

En zonas geográficas no endémicas de América, como también fuera del continente, se reportan casos en personas que nacieron, habitaron o visitaron zonas endémicas ^22^.

En la naturaleza, *Paracoccidioides* spp. se desarrolla como micelio y produce conidias infectantes que son transportadas por el aire después de la remoción de suelos. Estas conidias llegan a los alvéolos pulmonares del huésped susceptible y se convierten en estructuras levaduriformes, que pueden permanecen en los ganglios linfáticos y pulmonares. Las lesiones primarias pueden subsistir silenciosas durante muchos años y progresar en los pulmones o diseminarse por vía linfohematógena ^5^.

Las manifestaciones clínicas de la forma progresiva crónica de paracoccidioidomicosis ocurre en adultos, principalmente en hombres entre los 30 y los 50 años, que realizan actividades rurales. Ocasionalmente, en personas que vivieron o visitaron áreas endémicas de paracoccidioidomicosis. Se inicia como una neumonitis que puede extenderse por el sistema linfático a los ganglios, donde a menudo forma granulomas. Puede convertirse en una enfermedad latente, de evolución lenta y progresiva a lo largo de meses o años, capaz de reactivarse con manifestaciones unifocales o multifocales ^2^.

Una disminución de la respuesta inmune puede provocar la reactivación del microorganismo y la diseminación hematógena a diferentes tejidos y órganos. Los síntomas iniciales de la reactivación son inespecíficos. Las lesiones mucocutáneas con frecuencia son el motivo por el que los pacientes buscan atención médica ^16^^,^^19^ y se presentan como estomatitis ulcerosa.

Otros síntomas como la ronquera, la odinofagia, la disfagia, la faringitis, la disnea y las lesiones periodontales son manifestaciones clínicas prevalentes. Los pacientes también pueden presentar pérdida de peso, anorexia, tos, lesiones ulceradas en la orofaringe, y compromiso cutáneo, linfático, neurológico y suprarrenal ^2^^,^^9^^,^^16^^,^^19^.

Actualmente, se reconoce que el género *Paracoccidioides* es un complejo de especies filogenéticas: *P. brasiliensis sensu stricto* (S1), *P. americana* (PS2), *P. restrepiensis* (PS3), *P. venezuelensis* (PS4) y *P. lutzii*. La especie *P. brasiliensis* predomina en Brasil y Paraguay. En Argentina, las especies circulantes son *P. brasiliensis*, *P.restrepiensis* y *P. americana*. Recientemente, se describieron dos nuevas especies, no cultivables y asociadas a micosis subcutánea: *P. loboi* y *P. cetti*. (Teixeira MM, Cattana ME, Doyle A, Barker BM, Litvintseva A, Sosa MA, *et al*. Whole genome sequencing typing suggests multiple *P. brasiliensis* dispersion events into endemic areas of paracoccidioidomycosis in Argentina and Paraguay. 29° Congresso Brasileiro Microbiologia - CBM 2017- XIII International Meeting on Paracoccidioidomycosis, Foz de Iguazú, Parana, Brazil, 22-25 de octubre de 2017) ^19^^,^^21^^,^^23^^-^^26^.

Todas las especies del género se caracterizan por desarrollar, tanto en la naturaleza (15-30 °C), como *in vitro* (28 °C), un micelio hialino y tabicado con clamidioconidias y, raramente, conidias menores de 5 μm, llamadas propágulos. A temperaturas de 35 a 37 °C, en cultivos *in vitro* o en los tejidos del huésped susceptible, los propágulos se transforman en levaduras, multigemantes o no, de tamaño variable (4-40 μm) ^1^. La investigación sobre el dimorfismo térmico de *Paracoccidioides* spp. permitió comprender la versatilidad y la interacción entre las células fagocíticas del huésped y las conidias de este agente patógeno ^27^.

### Diagnóstico

Se define como criterio diagnóstico de caso probado todo paciente con manifestaciones clínicas compatibles con paracoccidioidomicosis e identificación de hongos levaduriformes, correspondientes a *Paracoccidioides* spp., en las muestras clínicas evaluadas mediante examen microscópico directo, con coloraciones o sin ellas, o recuperación del hongo en cultivo.

Por su parte, se establece caso probable cuando las manifestaciones clínicas del paciente son compatibles con paracoccidioidomicosis y presenta anticuerpos séricos específicos. En estos casos puede haber exposición previa al hongo teniendo en cuenta antecedentes de residencia, viaje a una zona geográfica endémica o excepcionalmente al contacto con fómites, como tierra o vegetación, procedentes de dichas zonas ^19^.

En el examen directo de las muestras clínicas se presenta *Paracoccidioides* spp. como una levadura con gemaciones simples o múltiples (forma parasitaria). En el examen microscópico de los cultivos (18-24 °C) idealmente se observa la forma típica de hifas hialinas tabicadas con microconidias tipo aleurioconidias, y ocasionalmente, clamidoconidias luego de 14 y hasta 40 días de incubación.

La observación microscópica de los exámenes directos requiere una buena calidad del espécimen clínico y un observador entrenado, en tanto que el cultivo requiere tiempo para identificar al hongo.

La detección de anticuerpos de *Paracoccidioides* spp. por técnicas serológicas presenta dificultades en cuanto a la falta de protocolos unificados y estandarizados para la producción de antígenos y antisueros. Actualmente, se reconoce que las diferentes especies del género *Paracoccidioides* pueden presentar antígenos inmunodominantes diferentes. Gp43, de 43 kDa, es el principal antígeno específico de superficie utilizado en pruebas serológicas que tienen una sensibilidad entre el 85 y el 100 %. Sin embargo, se han informado falsos negativos en pacientes infectados con *P. lutzii*, probablemente, por cambios genéticos de la especie que disminuyen la expresión de *gp43*, así como resultados falsos positivos por reacciones cruzadas con otros hongos ^28^^-^^31^.

Estas dificultades hacen que sea necesario incorporar técnicas moleculares, que complemenenten el diagnóstico micológico convencional, para identificar las especies de *Paracoccidioides* a partir de cultivos y detectar la presencia de ADN del género en los especímenes clínicos. A partir de muestras clínicas se utilizan: reacción en cadena de la polimerasa (PCR) simple, PCR doble, PCR anidada o semianidada, PCR en tiempo real (qPCR) y amplificación isotérmica mediada por bucles (LAMP).

Diferentes autores han llevado a cabo experimentos con PCRs anidada y semianidada para amplificar regiones espaciadoras internas transcritas (*Internal Transcribed Spacer*, ITS) y *gp43*, a partir de muestras clínicas, y han logrado buena sensibilidad y especificidad. Bialek *et al*. desarrollaron una PCR anidada para detectar *P. brasiliensis* mediante la amplificación de una región de *gp43*. Esta se evaluó con muestras de cultivos y tejidos de ratones infectados y obtuvieron alta especificidad analítica ^32^. Otros autores evaluaron esta PCR sobre muestras clínicas frescas o parafinadas para el diagnóstico de paracoccidioidomicosis ^33^^,^^34^.

En el presente trabajo demostramos que la detección de ADN de *Paracoccidioides* spp. en una PCR anidada, complementó el diagnóstico convencional de laboratorio de paracoccidioidomicosis en un hospital general fuera del área endémica ^31^.

El objetivo de este trabajo fue implementar la detección de ADN de *Paracoccidioides* spp. en el diagnóstico micológico convencional de pacientes con sospecha de paracoccidioidomicosis, atendidos en un hospital universitario de un área no endémica de esta micosis.

## Materiales y métodos

Se realizó un estudio retrospectivo con los datos del laboratorio de micología de un hospital universitario ubicado en la ciudad de Buenos Aires (Argentina) desde enero de 2015 hasta diciembre de 2022. Se incluyeron los resultados del examen directo, el cultivo, el serodiagnóstico por inmunodifusión y la detección de ADN de *Paraccoccidiodes* spp. de muestras de pacientes con sospecha clínica de paracoccidioidomicosis.

Se recolectaron los datos demográficos: edad, sexo, lugar de nacimiento y de residencia al momento de la consulta. Las muestras clínicas fueron procesadas para la observación del examen directo por microscopía y el cultivo según el procedimiento establecido por el Laboratorio de Micología del Hospital de Clínicas “José de San Martín” de la Universidad de Buenos Aires.

El examen se realizó con muestras en fresco, coloreadas con tinción de Giemsa y observadas con microscopia a 40 y 100 aumentos. Se sembraron dos juegos de tubos con agar Saboraud, Mycosel™ e infusión cerebro- corazón. Uno de los juegos se incubó a 28 °C y el otro a 37 °C durante 40 días. Se realizó la prueba de inmunodifusion en muestras de suero para detectar la presencia de anticuerpos anti- *Paracoccidioides* spp., con antígeno y antisuero control de la cepa *P. brasiliensis* B339, provistos por el Laboratorio Nacional de Referencia en Micología Clínica, Departamento de Micología del INEI - ANLIS “Carlos G. Malbrán”.

La extracción y la detección del ADN de *Paracoccidioides* spp. a partir de las muestras clínicas, se realizaron en el Servicio de Micosis Profundas del Departamento de Micología del INEI - ANLIS “Carlos G Malbrán”. En resumen, la extracción se realizó con el kit comercial, DNeasy Blood & Tissue Kit™ (QIAGEN) y la proteinasa K (QIAGEN), según instrucciones del fabricante, previo tratamiento de la muestra con liticasa, para degradación efectiva de la pared celular, y enzima proteolítica proveniente de *Thrichoderma harzianum*.

La detección de ADN de Paracoccidioies spp. se realizó por la técnica de PCR anidada, descrita por A. Bialek *et al*. ^32^, que amplifica un fragmento de 196 pares de bases del gen *gp43*. Esta prueba se llevó a cabo en el laboratorio de Micosis Profundas del Laboratorio Nacional de Referencia “Dr. Carlos G. Malbrán”.

## Resultados

En esta revisión, 9 de los 10 pacientes con paracoccidioidomicosis residentes en Argentina provenían o habían visitado zonas endémicas: seis provenían de países sudamericanos y para los tres restantes no se pudo determinar la procedencia geográfica. Solo un paciente, residente de la Provincia de Buenos Aires, expresó no haber visitado ninguna área endémica.

En el lapso de ocho años, se analizaron en el Laboratorio de Micología del Hospital de Clínicas 37 muestras clínicas con sospecha clínica de paracoccidioidomicosis de 19 pacientes adultos (17 hombres y 2 mujeres). Los pacientes habían nacido o habitaban, al momento de la consulta, en la región noreste de Argentina ^8^, Buenos Aires ^2^ Paraguay ^3^, Bolivia ^1^ y Perú ^2^. Tres pacientes no reportaron estos datos.

Las muestras procesadas fueron: ocho raspados de mucosa oral, ocho biopsias de laringe, cinco biopsias de piel, tres raspados de mucosa nasal, diez muestras respiratorias (lavado bronquial, lavado broncoalveolar, biopsia transbronquial, esputo), dos biopsias de ganglio y una biopsia de glándula suprarrenal. Se buscaron anticuerpos contra *Paracoccidioides* spp. en 15 sueros usando la técnica de inmunodifusión.

Se diagnosticó paracoccidioidomicosis en 12 de los 19 pacientes, con dos probables casos adicionales. Estos 14 pacientes con paracoccidioidomicosis probada y probable fueron hombres adultos con edad promedio de 53 años (rango: 23 a 72).

La observación microscópica directa evidenció levaduras multigemantes típicas, sugestivas de *Paracoccidioides* spp., en muestras de 11 pacientes y levaduras con morfologías aberrantes en un paciente ^35^. El hongo solo pudo ser recuperado en 4 de 12 pacientes con microscopía positiva; en uno de los cultivos se desarrolló la fase levaduriforme a 37 °C, mientras que en los otros tres se desarrolló la fase micelial a 28 °C. El serodiagnóstico detectó anticuerpos anti-*Paracoccidioides* spp. en ocho pacientes con paracoccidioidomicosis probada y en los dos pacientes con paracoccidioidomicosis probable (exámenes directos y cultivos negativos). Un paciente con examen directo positivo, en fresco y por observación histológica, no mostró anticuerpos anti-*Paracoccidioides* (cuadro 1).

**Cuadro 1:** Diagnóstico micológico de muestras clínicas de pacientes con sospecha de paracoccidioidomicosis

Paciente	Procedencia	Muestras	Examen directo	Histología	Cultivo	Serología	PCR	Paracoccidioidomicosis
1	Paraguay	Glándula suprarrenal, suero	LcP	NR	Positivo	Positiva	Positiva	Probada
2	Misiones	Suero	NR	NR	NR	Positiva	NR	Probable
3	Buenos Aires	Ganglio	Negativo	NR	Negativo	NR	Negativa	Negativa
4	Paraguay	Laringe, suero	Negativo	NR	Negativo	Positiva	Positiva	Probable
5	Misiones	Laringe	LcP	NR	Negativo	NR	Positiva	Probada
6	Entre Ríos	Esputo, suero	LcP	NR	Negativo	Positiva	Positiva	Probada
7	Misiones	Laringe, Paladar, suero	La	Positiva	Positivo	Positiva	Positiva	Probada
8	Paraguay	Piel	LcP	NR	Negativo	NR	Positiva	Probada
9	Buenos Aires	Laringe, suero	LcP	NR	Positivo	Positiva	Positiva	Probada
10	SD	Laringe, suero	Negativo	NR	Negativo	Negativa	Negativa	Negativa
11	Bolivia	Laringe, LB, suero	LcP	NR	Negativo	Positiva	Positiva	Probada
12	Chaco	Mucosa oral, suero	LcP	NR	Negativo	Positiva	NR	Probada
13	SD	Mucosa nasal, suero	Negativo	NR	Negativo	Negativa	NR	Negativa
14	Perú	Piel, suero	Negativo	Negativa	Negativo	Negativa	Negativo	Negativa
15	Formosa	Paladar	LcP	Positiva	Negativo	NR	NR	Probada
16	Entre Ríos	Piel, suero	Negativo	NR	Negativo	Negativa	Negativa	Negativa
17	Perú	Paladar, suero	LcP	Positiva	Negativo	Negativa	Negativa	Probada
18	Chaco	Mucosa oral, suero	LcP	Positiva	Negativo	Positiva	NR	Probada
19	Buenos Aires	Piel, BAL, suero	LcP	Positiva	Positivo	Positiva	Positiva	Probada

NR no remitida; BAL: lavado broncoalveolar; LB: lavado bronquial. LcP: levaduras compatibles con *Paracoccidioides* spp La: levaduras atípicas. SD: sin datos

En las muestras clínicas de 14 de los 19 pacientes se realizó PCR anidada para detectar ADN de *Paracoccidioides* spp. En 8 de los 9 pacientes con paracoccidioidomicosis probada, la PCR fue positiva, al menos, en una de las muestras clínicas, mientras que solo uno de los casos de paracoccidioidomicosis probable tuvo PCR positiva. Los cuatro pacientes restantes tuvieron resultados negativos en todas las pruebas realizadas y no se pudo diagnosticar paracoccidioidomicosis.

## Discusión

La paracoccidioidomicosis es una de las micosis endémicas más frecuentes en Latinoamérica y se caracteriza por un largo periodo de latencia ^2^^,^^15^^,^^16^. En área no endémica, como la Ciudad Autónoma de Buenos Aires, se reportaron casos de paracoccidioidomicosis de pacientes que vivieron o visitaron un área endémica y desarrollaron formas progresivas de la enfermedad. ^1^^,^^36^^,^^37^.

En un trabajo previo en el Hospital de Clínicas de la Universidad de Buenos Aires, con más de 350.000 consultas externas por año, se atendieron 18 pacientes adultos varones con paracoccidioidomicosis entre el 2002 y el 2017. El 61 % de estos pacientes provenía de área endémica, el 33,3 % había nacido en área endémica y migrado años atrás (entre 11 a 45 años) a Buenos Aires, y un paciente de la provincia de Buenos Aires manifestó no haber estado en área endémica (Fernández NB, Canteros C, Farias l, Toranzo A, Tiraboschi N, Stecher D. Paracoccidioidomicosis in a universitary hospital of Buenos Aires city. P092. 20th Congress of the International Society for Human and Animal Mycology. June 30 - July 4, 2020. Amsterdam, The Netherlands. https://doi.org/10.1093/mmy/myy036).

Todos los pacientes diagnosticados con paracoccidioidomicosis provenían de las áreas endémicas de Argentina y del resto de Sudamérica, excepto el paciente mencionado, residente de la Provincia de Buenos Aires, que manifestó nunca haber visitado las áreas endémicas del hongo. Entre el 2002 y el 2004, un estudio piloto realizado en zonas colindantes con el río Riachuelo, que limita el sur de la capital de Argentina, se identificaron dos sueros con anticuerpos anti-*Paracoccidioides* spp. en 141 perros estudiados. Los perros difícilmente se trasladan fuera de su lugar de nacimiento, por lo que de manera indirecta se registró la presencia del hongo en la zona (Canteros C., comunicación personal). Es probable que el área endémica de paracoccidioidomicosis se pueda estar extendiendo a zonas más australes de Argentina. Sin embargo, esto requiere la confirmación mediante estudios epidemiológicos y de detección de casos autóctonos.

El diagnóstico de paracoccidioidomicosis requiere un análisis crítico por parte de microbiólogos y clínicos, ya que no todas las pruebas diagnósticas están disponibles en los laboratorios de micología clínica en Latinoamérica. En una encuesta realizada por D. Falci *et al*., en 2018, entre laboratorios de la región y otros países de Latinoamérica (Brasil: 74 %; otros países, 26 %), se destacó que solo el 68 % de ellos hace observación microscópica (tratamiento con hidróxido de potasio) de las muestras clínicas, el 47 % de los centros realiza o deriva la detección serológica de anticuerpos contra *Paracoccidioides* spp. y muy pocos laboratorios utilizan herramientas moleculares para el diagnóstico de patógenos fúngicos; de ellos, el 59 % remite las muestras centros de referencia ^38^. Esta situación complica la capacidad de diagnosticar y tratar a los individuos con paracoccidioidomicosis, sumado a que el estado inmunológico del paciente y la manifestación de la enfermedad influyen en la eficacia del diagnóstico.

Los reportes de los resultados del examen directo de muestras de pacientes con paracoccidioidomicosis de áreas endémicas tienen sensibilidad variable. En un estudio de casos entre 1974 y 2008 en Botacatu, Brasil, se encontró que el 86 % de los exámenes directos fue positivo (345/401), mientras que, en otra área endémica, en el estado de Espirito Santo, entre 1978 y 2012, solo se hallaron levaduras sugestivas de *Paracoccidioides* spp. en el 30,7 % (168/546) de las muestras analizadas por examen directo ^5^^,^^7^^,^^39^.

En nuestro estudio, 12 de los 14 (85,7 %) pacientes con paracoccidioidomicosis fueron diagnosticados por examen directo en fresco, por estudios histológicos o ambos; en los dos pacientes restantes, no se observó el hongo en las muestras clínicas. Esto probablemente se deba a limitaciones en la toma de la muestra o a la baja carga fúngica. Una de las desventajas de la observación directa es que requiere, además de la experiencia del operador, muestras clínicas representativas y abundantes para poder observar las estructuras características de *Paracoccidioides* spp.

Otra dificultad en el diagnóstico de paracoccidioidomicosis es el difícil crecimiento de *Paracoccidioides* spp. *in vitro* y la probabilidad de contaminación por otros microorganismos de crecimiento más rápido, lo que hace que la recuperación en cultivo sea muy baja.

La temperatura es otro factor importante: aunque *Paracoccidioides* spp. puede crecer a 37 °C, esta no es su temperatura óptima de crecimiento sino de 25 a 30 °C, después de 2-3 semanas de incubación ^5^. También es importante considerar el uso de medios de cultivos específicos, como agar hecho con infusión cerebro corazón o con medio FavaNetto, que mejoran el rendimiento del cultivo. Por ejemplo, Hahn *et al*. obtuvieron un rendimiento del 97,1 % para el aislamiento de *P. lutzii* frente al 88,2 % de positividad en el examen micológico directo, utilizando estos medios de cultivos específicos ^19^. También Gonçalves Pinheiro *et al*. (2020) observaron una sensibilidad entre el 86 y el 100 % en los cultivos hechos a partir de muestras respiratorias y tejidos utilizando medio Fava-Netto ^30^. En nuestro trabajo, el hongo creció a partir de las muestras clínicas de cuatro (28,6 %) pacientes con paracoccidioidomicosis probada, probablemente varios de los factores enumerados estén relacionados con la baja recuperación del agente y, sobre todo, el hecho de que el medio enriquecido de Fava-Netto no se utiliza en la rutina de nuestro laboratorio.

Si bien el serodiagnóstico es una prueba clásica complementaria, esta posee una alta sensibilidad en el diagnóstico de paracoccidioidomicosis. En nuestro análisis, 10 de 11 pacientes con paracoccidioidomicosis probada o probable tuvieron anticuerpos contra el microorganismo. En dos pacientes con epidemiología y clínica compatible con paracoccidioidomicosis no se pudo observar el hongo, ni se obtuvo crecimiento en los medios de cultivo, pero la serología dio positiva. Inclusive, en uno de estos dos pacientes, se detectó ADN de *Paracoccidioides* spp.

Se evaluaron diferentes PCR para la detección de ADN de *Paracoccidioides* spp. Buitrago *et al*. utilizaron una PCR en tiempo real con ITS1 rDNA como blanco de amplificación, a partir de suero, sangre, esputo y biopsia de dos pacientes de áreas no endémicas ^40^. Rocha-Silva *et al*. realizaron una PCR en tiempo real amplificando una región del gen *pb27*, obteniendo alta sensibilidad y especificidad al evaluar el ADN contenido en muestras de sangre, suero, biopsia y lavado broncoalveolar ^41^. En 2022, A. Krakhecke-Teixeira *et al*. detectaron ADN de *Paracoccidioides* spp. mediante la amplificación por PCR en tiempo real de fragmentos del gen *gp43* y *hsp70* en 37 muestras biológicas (esputo, lavado broncoalveolar y muestras de tejido) ^20^.

La técnica molecular LAMP también ha sido analizada por otros autores, quienes amplificaron como blanco un fragmento del gen *gp43* a partir de muestras clínicas ^42^^-^^44^.

Pinheiro *et al*. (2021) evaluaron una PCR dúplex para diferenciar el complejo *P. brasiliensis* de *P. lutzii*. Los investigadores obtuvieron un límite de detección de 1 pg de ADN de *Paraccocidiodes* spp. y 100 % de especificidad, sin reacción cruzada con otros hongos patógenos ^28^.

En nuestro trabajo se utilizó una PCR anidada descrita por Bialek *et al*. ^32^ que dio resultado positivo en el 88,9 % de los casos de paracoccidioidomicosis probada y en uno de los casos probables. La detección de ADN de *Paracoccidioides* spp. permitió confirmar las morfologías atípicas observadas en el paciente 7, que luego fueron corroboradas por aislamiento del hongo, y complementar el diagnóstico con la serología positiva. Las formas atípicas o aberrantes fueron descritas por Restrepo en el 2000 para determinar si la morfología fúngica en los tejidos podría ser indicador de la viabilidad de las células de levadura de *P. brasiliensis*^35^; de igual manera, en el paciente 4, la PCR positiva complementó el serodiagnóstico positivo. En estos dos pacientes, definidos como paracoccidioidomicosis probable, puede que la escasa cantidad de carga fúngica en las muestras dificultara la observación microscópica.

En el paciente 11 se detectó ADN de *Paracoccidioides* spp. en una muestra respiratoria en fresco y en una biopsia parafinada de glotis. Cabe señalar que la detección de ADN a partir de tejido fijado e incluido en parafina es útil cuando el material no llega al laboratorio de micología para realizar examen directo y siembra de este, por lo cual, la PCR complementó el resultado de las técnicas histológicas.

En la PCR utilizada en este trabajo, amplificamos una región del gen *gp43* que codifica para una glicoproteína expresada por *Paracoccidioides* spp. Sin embargo, se conoce que existen diferencias epigenéticas entre las especies del complejo que pueden afectar el resultado de la PCR (falsos negativos) ^33^^,^^34^. Si bien la PCR demostró ser sensible y específica para el diagnóstico, al encontrarnos en un área no endémica, con amplio movimiento migratorio de población proveniente de áreas endémicas para *Paracoccidioides* spp., deberíamos incluir otros sitios blanco para poder detectar las distintas especies del género y mejorar la sensibilidad diagnóstica. Es importante recalcar que la toma de la muestra, su calidad, su adecuado transporte y su conservación son importantes en el laboratorio para aumentar la sensibilidad y especificidad diagnóstica.

En nuestro estudio retrospectivo, la implementación de una PCR anidada para detectar ADN de *Paracoccidioides* spp. complementó el diagnóstico convencional de paracoccidioidomicosis. Esta técnica molecular permitió demostrar la presencia del hongo cuando el examen directo fue negativo, cuando las estructuras microscópicas no fueron las esperadas o se necesitó esperar el tiempo del crecimiento fúngico para confirmar paracoccidioidomicosis, mejorando el diagnóstico micológico.

Hasta el momento solo se han evaluado unos pocos métodos moleculares *in house* sobre muestras clínicas que deberían continuar estudiándose, teniendo en cuenta la diversidad genética de las diferentes especies circulantes de *Paracoccidioides* spp. en la región.

La migración actual de poblaciones humanas por diversos motivos es un reto para el sistema de salud pública en Latinoamérica, que requerirá el acompañamiento de políticas públicas para atender una de las micosis desatendidas más prevalentes.
